# Occupational therapy and physiotherapy education and workforce in Anglophone sub-Saharan Africa countries

**DOI:** 10.1186/s12960-017-0212-5

**Published:** 2017-06-12

**Authors:** Augustine O. Agho, Emmanuel B. John

**Affiliations:** 1 Office of Academic Affairs, Old Dominion University, Norfolk, VA United States of America; 2 Department of Physical Therapy, Chapman University, Irvine, CA United States of America

**Keywords:** Occupational therapy education, Physiotherapy education, Sub-Saharan Africa, Health workforce

## Abstract

**Background:**

Sub-Saharan Africa (SSA) countries are faced with the challenge of educating a critical mass of occupational therapists (OTs) and physiotherapists (PTs) to meet the growing demand for health and rehabilitation services. The World Federation of Occupational Therapy (WFOT) and World Confederation of Physical Therapy (WCPT) have argued for the need of graduate-level training for OTs and PTs for decades. However, very few studies have been conducted to determine the availability of OT and PT training programs and practitioners in SSA countries.

**Methods:**

Initial data were collected and compiled from an extensive literature search conducted using MEDLINE and PubMed to examine the availability of OT and PT education and training programs in SSA countries. Additional data were collected, compiled, and collated from academic institutions, ministries of health, health professions associations, and licensing authorities in SSA countries. Secondary data were also collected from the websites of organizations such as the World Bank, World Health Organization (WHO), WFOT, and WCPT.

**Results:**

This investigation revealed that there are limited number of OT and PT training programs and that these training programs in Anglophone SSA countries are offered at or below the bachelor’s level. More than half of the countries do not have OT or PT training programs. The number of qualified OTs and PTs appears to be insufficient to meet the demand for rehabilitation services. Nigeria and South Africa are the only countries offering post-entry-level masters and doctoral-level training programs in physiotherapy and occupational therapy.

**Conclusions:**

Higher learning institutions in SSA countries need to collaborate and partner with other regional and foreign universities to elevate the educational training and increase the supply of PTs and OTs in the region.

## Background

Strategic initiatives and interventions for communicable diseases in Africa—especially in childhood—by WHO, governments, and non-governmental organizations from around the world, have shifted attention to the rise of chronic non-communicable diseases (NCD) as another emergent burden [1–6]. As people in sub-Saharan Africa (SSA) live longer, and its population gets older, the disease burden has been increasingly defined by chronic long-term care diseases and disabilities rather than premature mortality [4, 7–11]. While mortality and morbidity rates attributed to communicable diseases remain high, the prevalence of preventable non-communicable diseases has also increased significantly over the past five decades in countries around the world [3, 7, 8, 12]. According to WHO, 60% of deaths worldwide each year are attributed to accidents, injuries, and NCD such as coronary heart disease, hypertension, diabetes, stroke, and kidney disease [2]. Unfortunately, perennial shortage of the healthcare workforce on the African continent makes tackling chronic diseases and disabilities resulting from NCD challenging [13]. This is due in part to continued Africa population growth [10, 11] and migration of healthcare professionals to developed countries [14–16].

Rehabilitation in many of these non-communicable situations can be crucial, and studies reflect a lack of access to such rehabilitation in SSA [17–19]. Access to rehabilitation services for individuals with short- and long-term disabilities is lacking because of the inadequate number of qualified rehabilitation healthcare workforce, i.e., physiotherapists, occupational therapists, social workers, mental health counselors, speech therapists, and prosthetists and orthoptists [20, 21]. Interestingly, most of the studies of the healthcare workforce shortage in SSA have focused primarily on nurses and physicians [20, 21]. With the increasing number of people suffering and dying from preventable and treatable NCD and injuries in SSA, it is anticipated that the demand for qualified allied health professionals who provide rehabilitation services will increase [22, 23].

The unmet need for rehabilitation services can be addressed by recognizing physiotherapist (PT) and occupational therapist (OT) as major providers [19]. Individuals with movement dysfunction, functional limitations, and various disabilities due to illnesses, disorders, and amputations need the services of competent PT and OT [20]. PTs are trained to provide services that develop, maintain, and restore an individual’s maximum movement and functional ability across the lifespan. Movement dysfunction can be a result of aging, injury, diseases, disorders, conditions, or environmental factors [24]. Likewise, OTs are trained to promote the health and well-being of individuals through the therapeutic use of daily activities (occupations). The primary goal of OT is to enable people to participate in the activities of everyday life, achieving outcomes by working with individuals and communities to enhance their ability to engage in the occupations they want to, need to, or are expected to do or by modifying the occupation or the environment to better support their occupational engagement [25]. In studies from developed and some SSA countries, it was suggested that it would be advantageous to SSA countries facing critical shortage of healthcare workers to train and deploy OT and PT as mid-level healthcare practitioners who might be as effective as physicians for certain services [23, 26–30]. For instance, in South Africa, physiotherapists are recognized as autonomous healthcare professionals who can diagnose, treat, and refer patients to medical practitioners and specialists and order X-ray and other imaging studies, as well as issue a certificate of illness [23, 31].

OT and PT education varies greatly from country to country in SSA. WFOT and WCPT have supported efforts to increase and improve OT and PT education in sub-Saharan Africa by working with regional and national professional associations. To the best of our knowledge, few studies have attempted to systematically examine OT and PT educational programs and the workforce in SSA [32, 33]. While there is a need to examine the current capacity of all SSA countries to meet the demands for trained PT and OT, given resource constraints, data availability, and variation in health workforce training systems across the different language groupings, this study is only focusing on Anglophone SSA countries.

## Methods

An extensive literature search of relevant articles published in English Language within the last 10 years was conducted using MEDLINE and PubMed to identify from published papers whether OT and PT education and training exists in SSA countries. The electronic search was conducted between December 2015 and February 2016. The main search terms include: physiotherapy, occupational therapy, education, training level (i.e., diploma, bachelor, master, and doctoral), PT and OT professional organizations, health workforce, access to rehabilitation services, and licensing of PTs and OTs. For more focused search results, the terms “Africa”, and “Sub-Saharan Africa” were used with Boolean operators (AND, OR, and NOT). The literature searches were conducted by a research assistant (GRA), who at the time was a graduate student in a Master of Occupational Therapy entry-level program, and by one of the authors who is a physiotherapist. Relevant published papers retrieved using the specific search terms were reviewed by the authors. The title and abstract of relevant papers were first reviewed by the authors in order to determine whether full review and data extraction was necessary and appropriate. The GRA and the executive assistant to the primary author assisted in collecting and compiling secondary data collected from the websites of the following organizations: World Bank, WHO, WFOT, WCPT, Nigeria Physiotherapy Network, Occupational Therapy Africa Regional Group, Ghana Association of Physiotherapists, Zambia Association of Physiotherapists, and Health Professionals Council of South Africa. All the collated secondary data and information obtained from institutional websites were verified by seeking the feedback of PT and OT faculty members and key personnel at professional licensing/registration boards, professional associations, etc., located in SSA countries.

## Results

Table 1 presents the levels of occupational and physiotherapy programs offered in Anglophone SSA countries. Across Anglophone SSA countries, occupational therapy and physiotherapy training programs range from none to entry-level diploma and bachelor’s degree (B.Sc.) to academic doctoral degree (Ph.D.). Ethiopia is the only SSA country with a physiotherapy entry-level doctorate degree (DPT) training program. Sixteen (16) occupational therapy training programs were identified in Anglophone SSA. With the exception of the programs in South Africa, all of the occupational therapy programs are offered at or below the bachelor’s level. There are five academic master’s level and one doctoral level (Ph.D.) occupational therapy programs in South Africa. To the best of our knowledge, none of the higher learning (post-secondary) educational institutions in Botswana, Eritrea, Gambia, Lesotho, Liberia, Malawi, Namibia, Seychelles, Sierra Leone, Swaziland, and Zambia offer degree programs in occupational therapy.

**Table 1:** Occupational therapy and physiotherapy educational programs in Anglophone sub-Saharan Africa countries

Country	Institution	Occupational therapy				Physical therapy			
		Type of degree				Type of degree			
		Diploma	Bachelor’s	Master’s	Doctorate	Diploma	Bachelor’s	Master’s	Doctorate
Botswana	NA								
Cameroon	NA								
Ethiopia	Addis Ababa University								* X
	University of Gondar						X	X	
Eritrea	Asmara College of Health Science					X			
The Gambia	None								
Ghana	University of Ghana, Legion		X				X		
	University of Health and Allied Sciences						X		
Kenya	The Presbyterian University of East Africa, Kikuyu		X						
	Kenya Medical Training College, Nairobi	X							
	JKUAT, Nairobi		X				X		
	Moi University, Eldoret	X					X		
	Great Lakes University of Kisumu						X		
Lesotho	None								
Liberia	None								
Malawi	University of Malawi College of Medicine, Zomba						X		
Mauritius	University of Mauritius, Reduit, Mauritius		X				X		
Namibia	None								
Nigeria	Bayero University, Kano		X				X	X	X
	Federal School of Occupational Therapy, Oshodi, Lagos	X							
	Nnamdi Azikiwe, University, Nnewi						X		
	Obafemi Awolowo University, Ife		X				X	X	X
	University of Ibadan						X	X	X
	University of Lagos, Lagos						X	X	X
	University of Nigeria, Enugu Campus						X	X	X
	University of Maiduguri						X		
	University of Benin						X		
Seychelles	None								
Sierra Leone	None								
South Africa South Africa	University of Cape Town, Cape Town		X	X	X		X	X	X
	University of KwaZulu-Natal, Durban		X				X		
	University of Limpopo Medunsa Campus, Medunsa		X				X		
	University of Pretoria, Arcadia		X				X		
	University of Stellenbosch, Tygerberg		X	X			X		
	University of the Free State, Bloemfontein		X	X			X	X	X
	University of the Western Cape, Bellville		X	X			X	X	X
	University of Witwatersrand, Parktown, Johannesberg		X	X				X	X
Swaziland	None								
Tanzania	Tumaini University Kilimanjaro Christian Medical University College	X					X		
Uganda	Uganda Institute of Allied Health and Management Sciences	X				X			
	Mbarara University of Science and Technology		X				X		
Zambia	Lusaka Apex Medical University, Lusaka							X	
	University of Zambia						X	X	
Zimbabwe	University of Zimbabwe, Harare		X				X	X	

* X entry-level Doctorate of Physiotherapy, *NA* no data available

Twenty-six (26) physiotherapy educational programs exist in Anglophone SSA. Five universities (i.e., Obafemi Awolowo University, University of Ibadan, University of Lagos, University of Nigeria, and Bayero University, Kano) in Nigeria offer academic master and doctoral degrees in PT. Four universities (University of Cape Town, University of Free State, University of Western Cape, and University of Witwatersrand) in South Africa also offer master’s level and academic doctoral degrees in PT. With the exception of the programs in Nigeria and South Africa, all of the physiotherapy and occupational therapy programs are offered at or below the bachelor’s level. None of the higher learning educational institutions in Botswana, Gambia, Lesotho, Liberia, Namibia, Seychelles, Sierra Leone, and Swaziland offers degree programs in physiotherapy.

The number of occupational therapists and physiotherapists in Anglophone SSA are presented in Table 2. The information obtained from available sources indicates that the number of professional physiotherapists is higher than the number of professional occupational therapists in Anglophone SSA. Kenya and South Africa have the highest number of occupational therapists. Cameroon, Malawi, Mauritius, Swaziland, and Tanzania—countries with populations of 22.77 million, 16.7 million, 3.97 million, 1.27 million, and 51.82 million, respectively—have fewer than 100 physiotherapists and fewer than 100 occupational therapists. South Africa and Nigeria have the highest number of physiotherapists.

**Table 2:** Number of occupational therapy and physiotherapy professionals and educational programs in Anglophone sub-Saharan Africa countries

Country	Occupational therapy		Physiotherapy	
	# of entry-level programs	# of practitioners	# of entry-level programs	# of practitioners
Botswana	NA	NA	NA	NA
Cameroon	0	NA	0	102
Eritrea	0	NA	1	NA
Ethiopia	0^a^	NA	2	300
The Gambia	0	NA	0	NA
Ghana	1	4	2	200
Kenya	2	850	3	600
Lesotho	0	5	0	NA
Liberia	0	NA	0	NA
Malawi	0	10	1	92
Mauritius	1	49	1	90
Namibia	0	50	0	105
Nigeria	2	20	8	1250
Seychelles	0	4	0	NA
Sierra Leone	0	NA	0	5
South Africa	8	4 575	8	6 941
Swaziland	0	NA	0	23
Tanzania	0	96	1	41
Uganda	1	139	1	100
Zambia	0	5	2	370
Zimbabwe	1	124	1	320

Sources: World Confederation of Physical Therapy, 2017; World Federation of Occupational Therapy—Human Resources Project, 2012; Health Professional Council of South Africa (HPCSA); Ghana Society of Physiotherapy, Ghana Ministry of Health; Medical Rehabilitation Therapists (Registration) Board of Nigeria; Federal Ministry of Health, Nigeria

*NA* no data available

^a^A new occupational therapy program will be starting soon at the University of Gondar, Ethiopia [46]

## Discussion

The current study demonstrated that there is a shortage of rehabilitation healthcare workforce and limited number of occupational therapy and physiotherapy training programs in SSA. The findings of this study is in agreement with previous studies that indicated shortage of rehabilitation healthcare workforce [20, 23, 29, 34–36] and limited OT and PT training programs [32, 33, 35–38] in some SSA countries. For the past 65 years, WFOT and WCPT have been strong advocates for their respective professions [19, 36, 39, 40]. Both organizations have promoted international cooperation and collaboration and worked through their membership organizations in SSA to elevate/upgrade the level of training and education so that the practice and standards of services provided by practitioners can be advanced. The current study revealed that there are very few OT and PT educational programs and most of them are offered at or below bachelor’s level, resulting in a limited number of OT and PT healthcare providers who can be available to provide rehabilitation services [23, 29, 34, 35]. This suggests that there is an urgent need to strengthen and professionalize the local branches of PT and OT professional associations and for an expanded role of international organizations and collaboration among higher education institutions in Anglophone SSA countries in order to address the challenge of educating a critical mass of occupational therapists and physiotherapists and meet the growing demand for rehabilitation services. These findings are consistent with the results obtained in previous health workforce studies conducted in SSA [19, 36, 40]. For instance, Frantz reported in a study that lack of undergraduate training programs, limited number of therapists, need for upgrading knowledge base of physiotherapy educators, and recognizing physiotherapy as an essential service were some of the challenges facing physiotherapy education in Africa [36]. Recent studies by Balogun et al. suggests that, compared to many other SSA countries, Nigeria is probably positioned to play an important role in West African SSA region, in building capacity to train more OT and PT healthcare workforce and for international collaboration and exchanges [32, 33]. Similarly, results from the current study suggests that South Africa with the greatest number of OT and PT programs at academic master and doctorate degree levels is also better positioned as being a catalyst to build capacity for OT and PT healthcare workforce in the southern Africa region of SSA.

Unless a concerted effort is made to increase the number of OT and PT clinicians and educators with advanced degrees, the goal of developing and sustaining entry-level OT and PT educational programs at or above the bachelor entry-level will be difficult to achieve [20, 40]. The WCPT Africa Region in 2014 adopted a Vision 2020, with goals to help the few SSA countries currently operating diploma PT programs in Africa and to upgrade to bachelor’s degree level, and a Vision 2030, which will see upgrade of most bachelors entry level to clinical doctorate entry level [19]. The WCPT in various Guiding Policy documents has set international standards for entry-level education in physiotherapy as a minimum of Baccalaureate, Masters, or Doctoral entry-level education [41, 42]. The WCPT also now conducts international accreditation of educational institutions worldwide that wish to attain, maintain, and be recognized as meeting these international standards [43]. In addition, the WCPT has also been directly involved in taking steps to help upgrade education programs in some African countries. For example, through an international initiative to strengthen education programs in Africa, the WCPT in collaboration with Handicap International, and supported by funding from the United States Agency for International Development (USAID), will assist three African nations of Mali, Niger, and Senegal through the SUDA Project from 2016 through 2018 and beyond [44, 45]. The goals of SUDA are (1) strengthening three national physical therapy associations, (2) using WCPT standards to support three countries in improving physical therapy entry-level education in order to advance towards WCPT accreditation, (3) developing a WCPT policy paper related to physical therapy assistants (PTAs), (4) augmenting wheelchair training within the physical therapy sector. SUDA acronym is derived from each of the first letter of the four program objectives: S, U, D, and A [44, 45]. Through a similar international initiative, there are indications that Ethiopia will soon have its first occupational therapy training program, which will be established at the University of Gondar, Ethiopia. This will be made possible as part of a US$ 24.2 million grant to Queen’s University, Ontario, Canada (to be disbursed over a 10-year period), from The MasterCard Foundation [46]. The MasterCard Foundation maintains a network of 27 universities that supports accessibility in education, specifically for students with disabilities across the African continent [47]. Therefore, without similar international initiatives, cooperation, and partnerships among SSA country, these vision and goals may be difficult to achieve. Although extensive efforts were made to obtain OT and PT country data for SSA countries, the results of this study is limited by the inability of the authors to access current OT and PT country information for some SSA countries. Several SSA country data listed in Tables 1 and 2 were reported as “NA”, i.e., no data *available*.

### Recommendations for further studies

Given that the burden of injuries and non-communicable diseases (i.e., cardiovascular, cancers, diabetes and chronic lung diseases) is rising disproportionately among lower income countries and populations, the challenge of training individuals who can provide specialized rehabilitation (for example, post stroke and cardiac events) and activity of daily living services must be addressed. Nine suggestions are advanced for future research on OT and PT workforce in SSA. First, given that inadequate clinical fieldwork sites, lack of qualified faculty, and poor country-level awareness could potentially contribute to the low number of OT and PT educational programs and practitioners in SSA reported in this study, a future follow-up study should be expanded to include an assessment of the quality of the existing PT and OT educational programs. Studies similar to the recent report of Balogun et al. should be extended to other SSA countries [32, 33]. Second, this study did not investigate admission requirements, number of student enrolled, job placement, perception of new graduates, and employment settings of OT and PT practitioners. This should be considered in future studies. Third, given that the WFOT and WCPT have been advocating to upgrade the entry level into the OT and PT professions to beyond bachelor degree levels in all countries [36, 39], the feasibility of this initiative in developing countries should be assessed [33]. Fourth, the delivery of health and rehabilitation services is increasingly team based [48–50]. Research should be conducted to examine the extent to which the curriculum of existing PT and OT programs emphasize team-based and interdisciplinary education. Fifth, since physicians play a significant role in the delivery of healthcare and training of allied health professionals in SSA, an investigation of the views of physicians on the roles and contributions of PT and OT practitioners will provide critical information educators can use to design curriculum and strategies to establish OT and PT as integral members of the healthcare delivery team [51, 52]. Sixth, OT practice is generally based on western perceptions and classifications of activities of daily living and human occupation. The extent to which African perspectives are considered in the framing of the entry-level OT competencies should be examined [53]. Seventh, efforts should be made to identify and replicate effective models of international cooperation and partnership on the training and education of PT and OT [38, 40]. Eight, in order to obtain an accurate number of native-born and expatriate practicing PT and OT, an effort should be made to obtain information directly from professional licensing and government agencies. Finally, given resource constraints, data availability, and language constraints, this investigation focused on Anglophone SSA countries. This study should be expanded with specific focus on examining (a) the availability of OT and PT educational programs, (b) general knowledge and awareness of PT and OT professions among students, (c) employment settings for PT and OT, and (d) physician acceptance of the roles PT and OT in Francophone SSA.

## Conclusions

The study findings suggest that there is a paucity of rehabilitation healthcare workforce (specifically, OT and PT) in Anglophone sub-Saharan and limited number of training programs for OT and PT practitioners. Nigeria and South Africa have the highest number of PT training programs and PT practitioners, while South Africa and Kenya have the highest number of OT programs and OT practitioners. There is a need for inter-SSA collaboration to increase capacity of rehabilitation services workforce in SSA countries.
